# Neutrophil elastase downmodulates native G-CSFR expression and granulocyte-macrophage colony formation

**DOI:** 10.1186/1476-9255-7-5

**Published:** 2010-01-21

**Authors:** Melissa G Piper, Pam R Massullo, Megan Loveland, Lawrence J Druhan, Tamila L Kindwall-Keller, Jing Ai, Alexander Copelan, Belinda R Avalos

**Affiliations:** 1 Division of Pulmonary, Allergy, Critical Care, and Sleep Medicine, The Ohio State University, Columbus, OH, 43210, USA; 2 The Davis Heart and Lung Research Institute, The Ohio State University, Columbus, 43210, OH, USA; 3 Northeastern Ohio University College of Pharmacy, Rootstown, OH, 44272, USA; 4 The Division of Hematology and Oncology, The Ohio State University, Columbus, OH, 43210, USA; 5 Division of Cardiovascular Medicine, The Ohio State University, Columbus, OH, 43210, USA; 6 Department of Medicine, Comprehensive Cancer Center of Case Western Reserve University and University Hospitals of Cleveland, Cleveland, OH, 44106, USA; 7 Loyola University Chicago Stritch School of Medicine, Chicago, IL, 60660, USA; 8 Department of Internal Medicine, The Ohio State University, Columbus, OH, 43210, USA

## Abstract

**Background:**

The granulocyte colony-stimulating factor receptor (G-CSFR) plays a critical role in maintaining homeostatic levels of circulating neutrophils (PMN). The mechanisms modulating G-CSFR surface expression to prevent chronic neutrophilia are poorly understood. Here, we report that neutrophil elastase (NE) proteolytically cleaves the G-CSFR on human PMN and blocks G-CSFR-mediated granulopoiesis *in vitro*.

**Methods:**

Human peripheral blood PMN isolated from healthy donors were incubated with NE. Expression of the G-CSFR was analyzed by flow cytometry and western blot analyses. Detection of G-CSFR cleavage products from the culture supernatants was also performed. Human bone marrow mononuclear cells were also cultured in the presence or absence of NE to determine its effects on the proliferation of granulocyte-macrophage colony forming units (CFU-GM).

**Results:**

Treatment of PMN with NE induced a time-dependent decrease in G-CSFR expression that correlated with its degradation and the appearance of proteolytic cleavage fragments in conditioned media. Immunoblot analysis confirmed the G-CSFR was cleaved at its amino-terminus. Treatment of progenitor cells with NE prior to culture inhibited the growth of granulocyte-macrophage colony forming units.

**Conclusions:**

These findings indicate that in addition to transcriptional controls and ligand-induced internalization, direct proteolytic cleavage of the G-CSFR by NE also downregulates G-CSFR expression and inhibits G-CSFR-mediated granulopoiesis *in vitro*. Our results suggest that NE negatively regulates granulopoiesis through a novel negative feedback loop.

## Background

Granulocyte colony-stimulating factor (G-CSF) is the major regulator of granulopoiesis and supports the survival, proliferation, and maturation of myeloid progenitor cells along the neutrophil (PMN) lineage [1]. G-CSF also activates certain functions of mature PMN and stimulates hematopoietic stem cell mobilization [2-6]. The growth of neutrophilic granulocytes *in vitro *from progenitor cells committed to neutrophils and monocytes (CFU-GM) is absolutely dependent upon G-CSF and sigmoidally increases with increasing G-CSF concentrations [2,5,7,8]. A critical role for G-CSF in regulating granulopoiesis *in vivo *has been demonstrated in G-CSF null mice who have chronic neutropenia and severely impaired granulopoietic responses to infection [6].

The biological activities of G-CSF are mediated by the G-CSFR receptor (G-CSFR), a transmembrane protein predominantly expressed on the surface of cells of the neutrophil lineage [7]. Like other cytokine receptors, the extracellular portion of the G-CSFR binds ligand and the cytoplasmic tail transduces intracellular signals [3,4,7]. Studies of mice with knock-out or knock-in mutations in their G-CSFR gene have suggested the G-CSFR generates unique signals required for PMN production and marrow egress to maintain homeostatic levels of circulating PMN during basal and stress granulopoiesis [9-12].

G-CSFR null mice have chronic neutropenia, a uniform decrease in myeloid cells in the bone marrow, and defects in PMN activation [6,10]. Competitive repopulation assays in these mice indicate G-CSF drives nearly all of granulopoiesis under basal conditions and that G-CSFR signals regulate the *in vivo *production and maintenance of both committed-myeloid progenitor cells and primitive multipotential progenitors [12]. Additional insights have come from mice expressing a chimeric G-CSFR (GEpoR) comprised of the extracellular ligand-binding domain of the G-CSFR fused to the cytoplasmic domain of the erythropoietin receptor (EpoR) [13]. GEpoR mice retain the ability to produce PMN but have chronic neutropenia, and despite near normal bone marrow PMN levels, G-CSF treatment fails to mobilize significant numbers of PMN into the peripheral blood.

The regulated manner in which PMN are produced and released into the circulation suggests that positive regulation of granulopoiesis via G-CSF/G-CSFR interactions must be balanced by negative feedback loops [14,15]. However, little is known about the mechanisms downregulating G-CSFR surface expression to negatively regulate granulopoiesis. An *in vivo *role for neutrophil granule enzymes in both modulating PMN and stem cell mobilization and in downregulating G-CSFR surface expression on PMN was previously suggested by Jilma [16]. Subsequent studies by Levesque *et al *identified neutrophil elastase (NE) as a neutrophil granule enzyme that promotes stem cell mobilization by cleaving chemokines and chemokine receptors [17,18], such as stem cell derived factor-1 (SDF-1) and its corresponding receptor, CXCR4 [19].

Recent studies by our laboratory and others indicate that NE also degrades G-CSF and inhibits G-CSF-stimulated proliferative responses *in vitro *[20,21]. The paradigm of both ligand and receptor cleavage provided by SDF-1 and CXCR4 prompted us to investigate whether NE also cleaves the G-CSFR to modulate its expression and signaling and whether it might be the putative granule enzyme in PMN reported by Jilma that decreases G-CSFR surface expression [16]. Here, we show that NE proteolytically cleaves the G-CSFR to downregulate its expression on PMN and that it also inhibits G-CSFR-mediated granulopoiesis *in vitro*. These results suggest a novel role for NE as a negative regulator of granulopoiesis.

## Materials and methods

### Reagents and cell culture

Recombinant human G-CSF was generously provided by Amgen (Thousand Oaks, CA). Other cytokines were purchased from R&D Systems (Minneapolis, MN). Purified human NE was from Elastin Products Company, Inc. (Owensville, MO). Cathepsin G (CG) and azurocidin (AZ) were from Athens Research & Technology (Athens, GA). Phenylmethylsulfonyl fluoride (PMSF) and protease inhibitors were from Sigma-Aldrich Corp. (St. Louis, MO). Anti-G-CSFR antibodies recognizing the extracellular region of the human G-CSFR were obtained from BD Biosciences (Palo Alto, CA). Streptavidin-Cy5 was from Invitrogen (Carlsbad, CA) and biotinylated anti-streptavidin antibody from Vector Laboratories (Burlingame, CA). The polyclonal antibody recognizing the distal tail of the murine and human G-CSFR was obtained from Santa Cruz Inc (Santa Cruz, CA). Fetal bovine serum (FBS), RPMI media, and serum-free StemPro-34 media were from Invitrogen. Ba/F3 and COS-7 cells transfected with the full-length human G-CSFR cDNA, which have previously been described, were grown in RPMI media containing 10% FBS [22,23]. For culture of Ba/F3 cells, 10% WEHI conditioned media was also added as a source of interleukin-3 (IL-3).

### Flow cytometric analyses

PMN (97% purity) were isolated from peripheral blood of healthy human donors following appropriate informed consent using Ficoll-Hypaque (d = 1.077) and sedimentation in 3% dextran sulfate. Contaminating red cells were removed by lysis in an ammonium chloride solution (BD Biosciences). PMN (1 × 10^7 ^cells/ml) were incubated at 37°C with or without 0-150 μg/ml NE, AZ, or CG for varying times. Duplicate samples to which 10% serum or 1 mM PMSF was added during enzyme incubations were also included. Reactions were stopped by the addition of ice cold 10% FBS. G-CSFR expression on PMN was analyzed following fixation in 1% paraformaldehyde using immunofluorescence staining and a BD FACSCalibur Cytometer equipped with Cell Quest software (BD Biosciences) as previously described [21].

### Detection of proteolytic cleavage fragments of the G-CSFR

PMN and stably transfected Ba/F3 cells expressing the G-CSFR (1 × 10^7 ^cells/ml) were incubated with 150 μg/ml NE in PBS at 37°C for 0-120 minutes. Reactions were quenched by addition of 10% FBS and 1 mM PMSF. Samples were centrifuged and the supernatants containing the conditioned media collected. To detect G-CSFR cleavage fragments, conditioned media and whole cell lysates from the identical time points were analyzed by immunoblot analysis. Cell pellets were lysed as previously described [21], the samples (0.1 mg protein) loaded onto 10% polyacrylamide gels, transferred to nitrocellulose membranes, and immunoblotted.

#### Colony assays

Bone marrow was aspirated from the posterior superior iliac crest of healthy donors following appropriate informed consent. The bone marrow mononuclear cell fraction (BMMNC) was isolated using Ficoll-Hypaque. BMMNC were sequentially washed in RPMI 1640 containing 2 mM glutamine and 20% FBS followed by RPMI 1640 supplemented with 10% FBS and a final wash in StemPro-34 media. BMMNC (1 × 10^4^) were aliquoted into two fractions. One set was directly plated in StemPro-34 containing 1% methylcellulose (Stem Cell Technologies, Vancouver, BC, Canada), 1% BSA, 0.1 mM β-mercaptoethanol (2-ME), 2 mM L-glutamine, 10 μg/ml insulin, 200 μg/ml human iron-saturated transferrin, recombinant human insulin (10 μg/ml), G-CSF (20 ng/ml), stem cell factor (50 ng/ml), GM-CSF (20 ng/ml), IL-3 (20 ng/ml), IL-6 (20 ng/ml), and Epo (3 units/ml). The second set of BMMNC was pre-incubated with 0-2 μg/ml NE at 37°C for 90 min and washed to remove NE prior to plating in StemPro-34 media containing growth factors. The plated cells were incubated at 37°C for 14 days in a 5% CO_2 _humidified incubator and colony forming units (CFU)-GM enumerated using an Olympus CK2 inverted microscope equipped with a MagnaFire Digital Camera (Olympus America, Melville, NY).

## Results

### NE decreases G-CSFR surface expression on PMN

**Figure 1:**
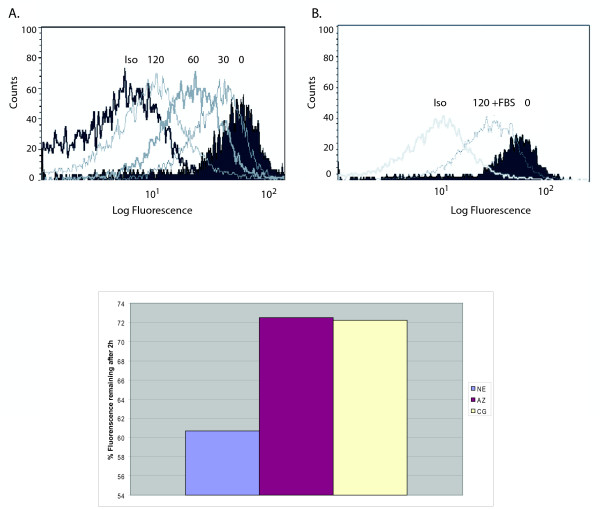
**Neutrophil elastase (NE) decreases G-CSFR expression on human PMN**. Peripheral blood neutrophils (PMN) from healthy donors (97% purity) were incubated (1 × 10^7 ^cells/ml) with 150 μg/ml NE at 37°C for the indicated times. G-CSFR surface expression was analyzed by flow cytometry as described using a biotinylated anti-G-CSFR antibody recognizing FNIII domains in the extracellular region of the G-CSFR (BD PharMingen) and a BD FACSCalibur Cytometer. A representative experiment from five independent experiments using neutrophils from five different donors is shown. **(A) **Effect of NE treatment for the indicated times on G-CSFR expression on PMN. Cells incubated with an isotype-matched control antibody (Iso) are shown as a negative control. **(B) **Effect of inclusion of 10% FBS (+) during incubation of PMN with NE. **(C) **PMN were treated with 150 μg/ml of NE, azurocdin (AZ), or cathepsin G (CG) for 2 h, then the G-CSFR was analyzed by flow cytometry. Shown is percent of fluorescence compared to maximum fluorescence at time 0.

We initially investigated whether treatment of freshly isolated PMN with NE altered surface expression of the endogenous G-CSFR. G-CSFR surface expression on PMN from healthy donors was analyzed before and after treatment of the cells with NE using flow cytometry (n = 5). As shown in Figure 1A, a time-dependent decrease in G-CSFR surface expression with near complete loss at 2 h was observed following treatment of PMN with NE.

To determine whether the decrease in G-CSFR surface expression in response to NE was enzymatically-mediated, 10% serum or 1 mM PMSF were included during the incubations. These concentrations of serum and PMSF have previously been shown to be sufficient to inhibit the enzymatic activity of NE [17,18,21]. As shown in Figure 1B, inclusion of 10% serum during incubation of PMN with NE markedly diminished the reduction in G-CSFR numbers observed in response to NE. Serum inhibited by 50% the reduction in G-CSFR surface expression observed after treatment of PMN with NE (Figure 1B). Similar results were obtained when PMN were treated with NE for shorter time periods and when PMSF was substituted for serum (data not shown). The diminished effect of NE on G-CSFR expression when serum or PMSF was added suggested the effect of NE was enzymatically mediated. We also examined the effect of other azurophilic granule proteases (CG and AZ) on G-CSFR expression over the same concentration range as NE using PMN from the same donors, and observed that only NE significantly decreased G-CSFR surface expression (data not shown).

### NE degrades the G-CSFR protein on PMN

**Figure 2:**
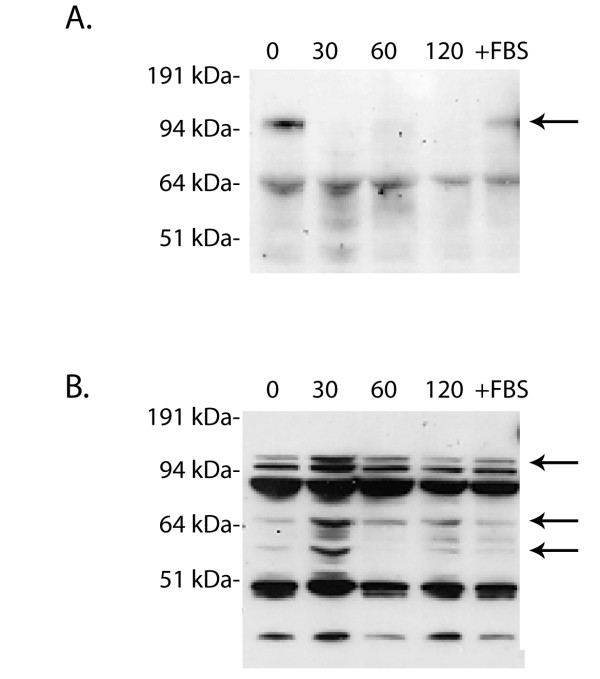
**NE proteolytically cleaves the G-CSFR on PMN**. PMN were treated with 150 μg/ml NE for the indicated times, the reactions quenched by addition of 10% FBS and 1 mM PMSF, and the cells lysed. Samples were resolved on 10% polyacrylamide gels, transferred to nitrocellulose, and the membranes immunoblotted with **(A) **the monoclonal antibody LMM741 recognizing FNIII domains in the extracellular portion of the G-CSFR (BD PharMingen). The arrow indicates the full length G-CSFR. **(B) **The blot in **(A) **was stripped then reblotted with rabbit polyclonal antibody recognizing the C-terminus of the G-CSFR (Santa Cruz). +FBS denotes lysates from PMN treated with NE for 120 min in the presence of 10% FBS. The arrows indicate the full length G-CSFR and resultant C-terminus generated by NE cleavage. A representative blot from three independent experiments (n = 3) is shown.

To investigate whether the decrease in G-CSFR numbers on PMN observed in response to NE was due to enzymatic degradation of the G-CSFR, we examined the G-CSFR protein in whole cell lysates. As shown in Figure 2A, a single band of M_r_~150 kDa corresponding to the molecular weight of the fully-processed human G-CSFR protein expressed at the cell surface was detected in immunoblots of whole cell lysates from untreated PMN using an antibody recognizing the FNIII domains in the extracellular region of the G-CSFR. Notably, when PMN were treated with NE, this band became undetectable by 30 min, and remained undetectable after 2 h. When 10% serum and 1 mM PMSF were included during the incubations with NE, the ~150 kDa band did not disappear and could still be detected at 2 h (Figure 2A, last lane), similar to PMN that were not treated with NE.

In order to determine whether the inability to detect the ~150 kDa receptor band on NE-treated PMN was due to a loss of the antibody recognition site as a result of enzymatic degradation, the blot in Figure 2A was stripped and re-blotted with an antibody recognizing the cytoplasmic tail of the G-CSFR. Immunoblot analysis of whole cell lysates from untreated PMN using this antibody yielded multiple bands, including the 150 kDa mature G-CSFR protein and a faint ~130 kDa band, two prominent bands in the 80-110 kDa range, and two faint lower molecular weight bands (Figure 2B). When PMN were treated with NE, the 150 kDa band could no longer be detected at 30 min and remained undetectable throughout the duration of the 2 h incubation. The intensity of the lower molecular weight bands increased as the incubation time with NE increased and appeared to correlate with the disappearance of the 150 kDa band corresponding to the mature G-CSFR protein. In addition, this antibody detected a new ~50 kDa band in PMN treated with NE which could not be detected at any time point examined in untreated PMN. The new band was detected within 30 min and the intensity of this band increased with longer incubation periods over the 2 h time period examined. The presence of 10% FBS completely inhibited the appearance of the new band and disappearance of the mature 150 kDa receptor species (Figure 2B, last lane). These findings are consistent with proteolytic degradation of the G-CSFR by NE. Furthermore, the inability to detect the mature form of the G-CSFR protein on NE-treated PMN with an antibody recognizing the extracellular region of the G-CSFR and the ability to detect a new 50 kDa band in PMN following treatment with NE solely with an antibody recognizing the cytoplasmic tail of the G-CSFR support a mechanism whereby the G-CSFR is degraded by NE via proteolytic cleavage at a site within the extracellular portion of the G-CSFR.

### Detection of G-CSFR cleavage fragments in conditioned media

**Figure 3:**
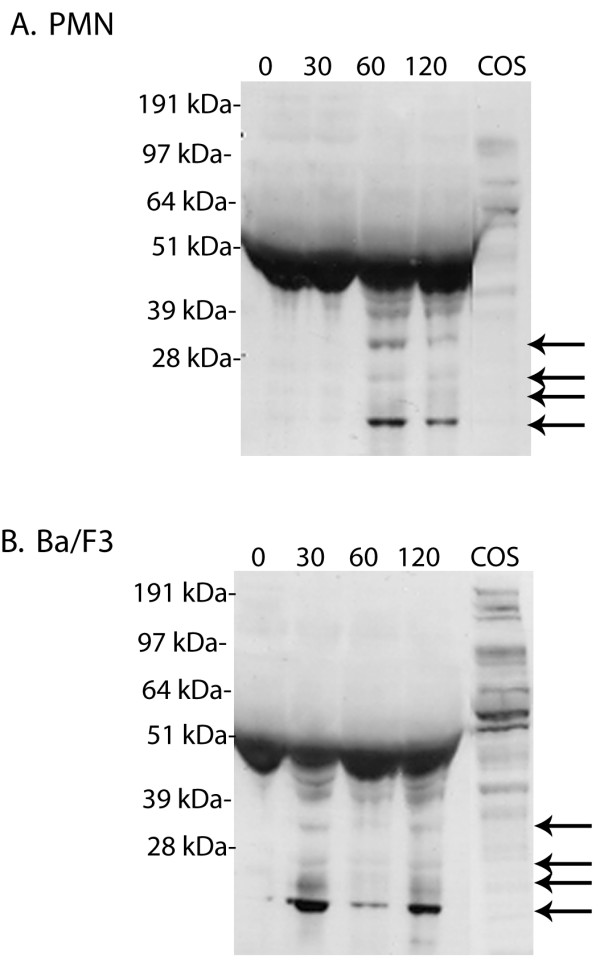
**Detection of G-CSFR cleavage fragments in conditioned media from NE-treated cells**. **(A) **PMN and **(B) **Ba/F3 cells transfected with the full-length G-CSFR cDNA were treated (1 × 10^7^cells) with 150 μg/ml NE for the indicated times. Reactions were stopped by the addition of 10% FBS and 1 mM PMSF, the samples centrifuged, and the supernatants containing the conditioned media collected and immunoblotted with an antibody recognizing the FNIII domains in the extracellular region of the G-CSFR. Arrows indicate the extracellular G-CSFR fragments generated by NE. A representative blot from three independent experiments (n = 3) is shown.

We next analyzed conditioned media from untreated and NE-treated PMN for the presence of G-CSFR cleavage fragments to obtain confirmatory evidence that NE proteolytically cleaves the G-CSFR. Proteolysis within the membrane-exposed portion of the G-CSFR is predicted to result in the generation of cleaved fragments of the extracellular portion of the G-CSFR that are released into the conditioned media and detected as lower molecular weight bands that should only be apparent after NE treatment. As shown in Figure 3A, immunoblot analysis of the conditioned media collected from the same PMN from which whole cell lysates were examined in Figure 2 using the antibody recognizing the extracellular portion of the G-CSFR detected the presence of multiple lower molecular weight bands of M_r _< 50 kDa following treatment of PMN with NE. Although a prominent non-specific band of M_r _~50-55 kDa was seen at all time points in the conditioned media both before and after NE-treatment, immunoreactive bands of M_r _< 50 kDa could only be detected in media harvested from PMN that were treated with NE. Since the extracellular domain of the G-CSFR is predicted to have a M_r _~60 kDa, detection of bands smaller than this only after treatment of PMN with NE supports a mechanism involving cleavage of the G-CSFR in its extracellular region. As expected, immunoblot analysis of whole cell lysates from untreated COS-7 cells transfected with the full-length G-CSFR cDNA (Figure 3A, last lane) and of untreated PMN (Figure 2A) using the same antibody yielded a 150 kDa band consistent with the mature membrane-anchored G-CSFR protein [23,24]. The appearance of the multiple lower molecular weight G-CSFR species in conditioned media from NE-treated PMN (Figure 3A) correlated with the time course observed for degradation of the membrane-anchored G-CSFR by NE (Figure 2B). Proteolytic cleavage fragments of the G-CSFR in the conditioned media of NE-treated PMN were apparent within 60 min, at a time when degraded forms of the G-CSFR protein could also be detected in whole cell lysates from the same PMN samples treated with NE.

Since we previously showed that in stably transfected Ba/F3 cells the ectopically expressed G-CSFR is cleaved by NE [21], we also examined conditioned media from these cells for the presence of cleavage fragments of the G-CSFR. As shown in Figure 3B, treatment of Ba/F3 transfectants with NE resulted in the appearance of multiple lower molecular weight G-CSFR cleavage fragments in the conditioned media. Bands of M_r _< 50 kDa were detectable in the conditioned media by 30 min, a time slightly earlier than their appearance in conditioned media from NE-treated PMN.

### NE inhibits G-CSFR-mediated CFU-GM growth

**Figure 4:**
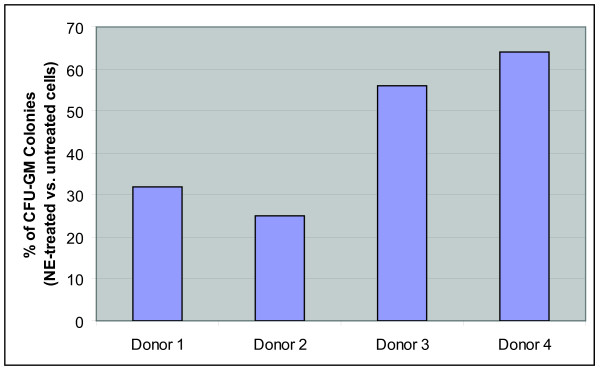
**Pretreatment of bone marrow-derived myeloid progenitors with NE inhibits granulocyte colony formation**. Bone marrow mononuclear cells (BMMNC) isolated from four healthy donors were left untreated or treated with 1 μg/ml NE for 90 min at 37°C. The cells were then washed, resuspended in StemPro-34 serum-free media, and plated (1 × 10^4 ^cells) in serum-free Methocult containing 1% methylcellulose, G-CSF (20 ng/ml), and a cocktail of recombinant growth factors as described in the *Materials and Methods*. CFU-GM were enumerated after 14 days. Data are represented as the percent of CFU-GM colonies arising from NE-treated cells compared to untreated cells.

Since the growth of CFU-GM *in vitro *has been shown to be absolutely dependent upon G-CSF, which transduces signals through the G-CSFR [7,15], we investigated the effects of treatment of myeloid progenitor cells with NE on the signaling function of the G-CSFR. For these studies, the effect of NE on the growth of myeloid progenitor cells purified from the mononuclear cell fraction of bone marrow (BMMNC) from healthy human donors was examined (n = 4). Pre-treatment of BMMNC with NE prior to their culture in methylcellulose supplemented with G-CSF and other growth factors reduced CFU-GM growth by as much as 75% (Figure 4). The inhibitory effect of NE on CFU-GM growth was also observed to be dose-dependent (data not shown).

## Discussion

A remarkable feature of granulopoiesis is the regulated production and release of PMN to maintain homeostatic levels in the circulation during basal granulopoiesis and to rapidly increase numbers during environmental stress [15]. G-CSF is the major cytokine regulating granulopoiesis [3,6,7], and its regulatory capacity depends upon its ability to bind to the G-CSFR. Thus, both G-CSF concentration and G-CSFR numbers modulate myeloid cell responsiveness and, hence, PMN numbers. The physiologic processes that regulate G-CSF levels have been well-characterized [7], but little is currently known about the mechanisms modulating G-CSFR surface expression.

Ligand-binding has been shown to trigger endocytosis and internalization of most growth factor receptors, which are then either recycled back to the cell surface or degraded intracellularly [3,24]. Ligand-induced internalization decreases the number of surface receptors and thereby serves to attenuate growth factor-induced signals [25-27]. In the case of the G-CSFR, ligand binding has been shown to modulate G-CSFR surface expression *in vitro *[23,28]. Following ligand binding, the G-CSFR on immature myeloid cells, U937 cells, and PMN is rapidly internalized and degraded [28]. More than 70% of specifically bound G-CSF is internalized after 5 min. Treatment of PMN with GM-CSF, TNF, LPS, fMLP, TPA, or C5a also downregulates G-CSFR numbers, while only TPA significantly reduces G-CSFR numbers on immature cells. There is no evidence the G-CSFR is recycled back to the cell surface.

Studies of naturally-occurring G-CSFR deletion mutants isolated from patients with severe congenital neutropenia (SCN) transforming to acute myelogenous leukemia (AML) have provided evidence for the importance of downregulation of G-CSFR expression [29,30]. A critical cytoplasmic domain that mediates G-CSFR internalization and degradation has been shown to be deleted in cells from these patients, which exhibit enhanced growth and survival signals to G-CSF [21,23,31,32]. G-CSFR surface expression is prolonged and G-CSF-mediated activation of Stat5 and Akt are sustained in these cells, indicating that receptor downregulation plays a critical role in extinction of G-CSFR signals.

There is also evidence that the G-CSFR on PMN is downregulated *in vivo *in response to G-CSF. Jilma *et al *showed that a single injection of G-CSF decreased G-CSFR numbers on PMN in humans by ~75% [16]. These effects were noted as early as 6 min, peaked at 90 min, and did not return to pre-treatment levels for 2 days. Notably, PMN numbers were transiently decreased and plasma levels of the neutrophil granule enzyme gelatinase b (also known as matrix metalloproteinase-9 or MMP-9) were increased 10-fold after G-CSF administration, implying a role for gelatinase b in both decreasing G-CSFR levels and PMN numbers. In related studies of the *in vivo *effects of lipopolysaccharide (LPS) infusion on G-CSFR expression, a significant negative correlation between PMN activation and G-CSFR expression was found [33]. The effects were reported to be independent of LPS-induced increases in G-CSF levels *in vivo*, suggesting that PMN activation and G-CSFR expression are tightly co-regulated.

In the current paper, we have identified an alternative mechanism for modulating G-CSFR expression on PMN involving the primary granule enzyme NE. Our data provide the first evidence that NE cleaves the endogenously expressed G-CSFR on PMN and inhibits G-CSFR-mediated granulopoiesis *in vitro*. Treatment of PMN with NE induced a time-dependent reduction in G-CSFR surface expression and the appearance of G-CSFR cleavage fragments in conditioned media from treated PMN. Both serum and PMSF could prevent degradation of the G-CSFR, suggesting NE degrades the G-CSFR by enzymatic cleavage. The time-course for the appearance of G-CSFR cleavage fragments in conditioned media from NE-treated PMN correlated well with the decrease observed in G-CSFR surface expression on treated PMN as detected by flow cytometry. We also show that NE abrogates proliferative signals generated by the G-CSFR in myeloid progenitor cells, as indicated by the decreased number of CFU-GM arising from NE-treated marrow progenitor cells. Our data demonstrate that NE cleaves the G-CSFR at a site within its extracellular portion, within which lies the ligand-binding site for G-CSF. Notably, proteolytic cleavage of the G-CSFR within this region is predicted to modify the binding site for G-CSF and thereby affect the sensitivity of cells to G-CSF, consistent with our data.

We [21]and others [20] have previously reported that NE also cleaves G-CSF and antagonizes its *in vitro *activity. However, unlike El-Ouriaghli *et al *who could not demonstrate an effect of NE on the G-CSFR [20], we reported that NE could also cleave the G-CSFR on transfected Ba/F3 cells [21]. It is possible that El-Ouriaghli's group failed to observe an effect on the G-CSFR due to the significantly longer period of culture (up to seven days) in NE-containing media they used before analyzing G-CSFR expression or because of the lower pH (5.5 vs.7.2) of their reconstituted NE.

NE has been reported to cleave multiple substrates in addition to G-CSF and the G-CSFR including SDF-1, CXCR4, VCAM-1, CD14, CD23, and complement receptor 1 (CR1) [17-19,34-37]. For many cytokine and chemokine receptors that are cleaved by NE, the extracellular ligand binding region is the site of cleavage [17-19,36,38]. Our findings with NE and the G-CSFR suggest a similar role for NE in regulating both cytokine and its respective receptor levels as reported for SDF-1 and CXCR4 [19]. Inactivation of SDF-1/CXCR4 interactions by NE was shown to induce hematopoietic stem cell mobilization. In G-CSF-induced stem cell mobilization, NE levels increase *in vivo *in the plasma and bone marrow microenvironment where PMN accumulate [17,36,39].

More recently, NE was reported to downregulate expression of c-KIT (CD117), the receptor for SCF [18]. Decreased c-KIT expression and increased NE levels were demonstrated in the marrows of mice receiving G-CSF for stem cell mobilization. Similar to our findings with the G-CSFR, a 2 h incubation with NE was required to induce a 50% reduction in c-KIT surface expression.

During G-CSF-induced stem cell mobilization, plasma levels of NE dramatically increase reaching levels of approximately 1 mg/ml. Within individual azurophilic granules from activated PMN, concentrations of NE in excess of 5 mM (150 mg/ml) have been measured. Thus, the concentrations of NE (0-150 μg/ml) and serum (a source of alpha-1 anti-trypsin) used in our experiments are well within the ranges reported *in vivo *[40-44].

The functional significance of our findings *in vivo *remains speculative, as we did not directly examine this. However, NE-induced downregulation of G-CSFR expression *in vivo *could promote cellular egress or inhibit further expansion of the myeloid compartment. A possible scenario is that during stem cell mobilization, release of NE from accumulating PMN in the bone marrow functions to inhibit granulopoiesis by degrading the G-CSFR and thereby preventing progressive and uncontrolled neutrophilia.

Our findings may have particular relevance to understanding the pathogenesis of SCN. In the majority of patients with this disease, mutations in the ELA2 gene encoding NE have been identified, some of which result in aberrant targeting of NE to the plasma membrane [45]. It is possible that such NE mutants induce aberrant and/or accelerated cleavage of the G-CSFR in some cases of SCN.

## Conclusions

Our findings add the G-CSFR to the growing list of hematopoietic cytokine/chemokine receptors regulated by NE, and suggest a novel pathway for regulating G-CSFR levels at the cell surface in addition to transcriptional controls and ligand-induced internalization. Cleavage of both G-CSF and the G-CSFR could provide an additional mechanism for fine-tuning PMN numbers. Future studies in humans to examine G-CSFR levels on cells in the blood and marrow before, during, and after stem cell mobilization, and during periods of environmental stress such as infection, may help to clarify the *in vivo *relevance of NE-induced cleavage of the G-CSFR.

## Abbreviations

G-CSF: Granulocyte colony-stimulating factor; G-CSFR: granulocyte colony-stimulating factor receptor; NE: neutrophil elastase; CG: cathepsin G; AZ: azurocidin; BMMNC: bone marrow mononuclear cell fraction; CFU-GM: colony forming units-granulocyte macrophage; IL: interleukin.

## Competing interests

The authors declare that they have no competing interests.

## Authors' contributions

MGP wrote the manuscript, designed and performed experiments. PM, LD, and TK assisted in experiments. ML and AC contributed to data analysis. BRA designed experiments and wrote the manuscript. All authors read and approved the final manuscript.
